# Adult Patient Perspectives on Psychiatric Polygenic Risk Scores

**DOI:** 10.64898/2026.09.24.26363942

**Published:** 2026-09-27

**Authors:** Craig W McFarland, Amanda Merner, Page M Trotter, Abigail C Martinez, Lauren A. Ginn, Jonathan Frumovitz, Daphne M Ayton, Takahiro Soda, Eric A W Storch, Stacey Pereira, Gabriel Lazaro-Munoz

## Abstract

Polygenic risk scores (PRS) are rapidly developing, enabling estimates of genetic risk for developing psychiatric disorders. Psychiatric PRS are also increasingly entering consumer and clinical settings. Yet, little is known about how adults with psychiatric disorders perceive their potential use. Here, we conducted semi-structured interviews with 29 adults with psychiatric disorders to examine their perspectives on hypothetically receiving psychiatric PRS for themselves and their current or future children. Key themes emerged around perceived utility, motivations and deterrents to genetic testing, interpretation of PRS, and anticipated responses to results. In particular, participants expressed hope that PRS could support self-understanding, inform better psychiatric care, and enable earlier recognition of difficulties for their children. Participants however raised multiple concerns, including those of privacy, stigma, discrimination, eugenics, and the risk of self-fulling prophecies. Together, these findings offer critical empirical evidence to inform guidance on the responsible use of psychiatric PRS in clinical and consumer settings.

## Full Text Availability

The license terms selected by the author(s) for this preprint version do not permit archiving in PMC. The full text is available from the preprint server.

